# High-Cost Healthcare Technology Versus Employment Generation: Equity, Evidence, and Influence in Developing Health Systems

**DOI:** 10.7759/cureus.104649

**Published:** 2026-03-04

**Authors:** Habib Md R Karim

**Affiliations:** 1 Anesthesiology, Critical Care, and Pain Medicine, All India Institute of Medical Sciences, Guwahati, IND

**Keywords:** advanced health tech, affordable healthcare, developing countries, healthcare simulation, pneumatic transport system, robotic surgical platforms

## Abstract

Many low- and middle-income countries are spending money on high-tech healthcare, such as simulation centres, pneumatic tube systems, and even robotic platforms. These upgrades are sold as a sign of modernisation and global parity. Yet, people in these places pay a lot out of pocket for basic care, and many end up broke from catastrophic healthcare costs. Notably, most of these technologies do not always deliver real patient-level benefits. They are often underused, incur substantial maintenance, and are usually installed in tertiary care hospitals, leaving the majority of the population behind. When governments spend a substantial amount on these advanced technologies, they have less left towards workforce expansion, creating allied jobs, or helping people actually afford care. Importantly, a significant portion of these investments goes underutilised, limiting their intended system-level impact. In the resource-constrained economies where every dollar counts, what matters more right now is setting the right priorities. Although the digital health and pursuit of high-quality healthcare remain essential, strategic emphasis on core services and investments that strengthen the workforce and generate employment opportunities is equally important for building resilient and sustainable health systems for all. It is not that advanced healthcare technologies are not of any value, but the adoption should be when patient-level value and system feasibility are demonstrated. Further frugal innovation plays a great role in the advancement of health technology and digital health implementation. Nevertheless, low cost does not necessarily mean low impact. Thus, in the absence of robust data on the patient-level long-term outcome benefits, its adoption should be context-sensitive and supported by good evidence on patient outcomes and survival, over and above safety. High-cost health technology investments should be assessed through the lens of cost-effectiveness. The present editorial argues a prioritisation framework balancing equity, evidence, and opportunity cost in the light of digital health, frugal technology adoption in place for high-cost health technologies where feasible, and employment generation.

## Editorial

Over the last few years, many developing countries have invested significant funds in high-cost healthcare technologies, such as simulation centres, pneumatic tube systems (PTS), and robotic surgery platforms. These investments are often highlighted as steps towards modernising the health system and aligning it with global standards. While this narrative sounds good, it obscures a crucial question: do such technologies truly address the core problems patients face? Whom do they primarily benefit?

While these questions matter everywhere, they are far more relevant in low- and middle-income countries (LMICs) where per capita expenditure on health is very low [1]. Health systems of LMICs always operate under stretched budgets [1]. Even the fiscal capacity and financing models vary across different countries. Nevertheless, budgeting models, including performance-based financing, should be reliant on each country’s infrastructure and conditions [2]. Public spending on health stays limited, unemployment is high, and patients still pay a significant share of their care out of pocket [3]. In many LMICs, out-of-pocket (OOP) payments continue to dominate healthcare financing and remain a major cause of catastrophic health expenditure (CHE) [4,5]. For a lot of families, a single hospital visit can wipe out savings, make them sell their livestock and properties, and push them into poverty. A recent Indian sample has shown that OOP expenditure adds 2.61% to poverty, which corresponds to 6.47 million households [6]. A systematic review shows that nearly 43-47% of healthcare expenditure is OOP despite an increase in government spending over the last decade [7]. Utilisation of private healthcare facilities, larger households, and chronic illnesses that are predominantly found in LMICs increase the odds of impoverishment [6].

While outpatient care is costly, inpatient care is where those financial shocks are felt most sharply. In India, for example, evidence shows that hospitalisation of children under five frequently leads to CHE [8]. The drivers are not just long hospital stays or expensive investigations and drugs; travel, food, a place to stay, and lost wages when a parent or family member has to take time off work also need significant consideration. Medical care for older persons also faces similar risks of CHE [9]. The role played by the publicly funded insurance schemes and subsidised healthcare is crucial. Large government-funded insurance schemes such as Ayushman Bharat-Pradhan Mantri Jan Arogya Yojana (PM-JAY) and preventive programs like Rashtriya Bal Swasthya Karyakram (RBSK) of India are notable. They have undoubtedly improved access to health care and provided some degree of protection against financial bankruptcy. But lots of families still fall through the cracks of these schemes. The reality is, there are significant coverage gaps, and hidden costs keep piling up [10,11]. Complete free healthcare or 100% coverage under such programs by the public fund is nearly impossible in LMICs. On the other hand, insurance coverage is minimal and has significant gaps across the LMICs, ranging from 7.9% in low-income and 27.3% in lower-middle-income countries [12]. Private health insurances are far lower [13]. It is largely due to limited knowledge or understanding of the insurance concept and affordability concerns. Further, inconsistent or incomplete coverage, eligibility policies, preferential allotment, geographic limitations of covered hospital networks, and their travel costs significantly affect the adoption and retention of health insurance policies in LMICs [14]. Notably, that is not just an Indian problem. It is happening in other LMICs with similar insurance schemes [12-15].

It is against this background that governments' decisions involving substantial expenditure on high-cost infrastructure warrant careful and detailed evaluation, with explicit consideration of the potential direct trade-offs for healthcare delivery, resource allocation, and system sustainability. There is a growing emphasis on tertiary care and digital health within contemporary healthcare budgets, reflecting an important and necessary evolution in service delivery. However, dedicated standalone allocations for high-cost healthcare technologies remain uncommon. Even when such provisions are present, they are typically incorporated within the broader health budget, which is already constrained by competing priorities. As a result, each major funding in advanced healthcare technology draws from a finite resource pool, underscoring the need for thoughtful prioritisation and careful stewardship of available funds, especially in the public sector. While in the private sector, such investments are often weighted against return on investment (RoI), which ultimately shifts the cost burden to patients' pockets and thus increases the OOP expenditure and risks for CHE, the RoI is not usually considered in the public sector, which usually runs at the subsidised price and results in a loss in terms of revenue generation. Thus, it can divert funds from other critical priorities, such as recruiting healthcare professionals, strengthening district hospitals, or creating allied health jobs within the tertiary healthcare infrastructure as well. From a health economics perspective, this trade-off raises concerns about allocative efficiency, as limited resources potentially get diverted from interventions that could deliver broader and fairer benefits [15].

And then there are equity concerns, which are even harder to ignore. Public health spending, at least in principle, should prioritise those with the greatest need. In reality, however, high-cost technologies tend to be concentrated in tertiary hospitals, which are mostly located in cities and serve relatively small, often wealthier patient populations. For example, nearly 75% of the infrastructure is distributed in urban areas, which have 27% of the population [16]. Further, tertiary care hospitals like medical colleges, corporate hospitals, and institutes are mostly located in cities or district headquarters. Although some gap is expected due to the hierarchy of the healthcare system structure, evidence shows that place of residence strongly determines the healthcare utilization [17,18]. Although governments are improving rural healthcare, an urban-rural gap of 41% utilisation is a big concern. Thus, the huge gap in urban-rural high-cost health technologies creates an ethical tension. The government's effort to improve the tertiary healthcare and digital health is essential and requires a high cost. However, not all high-cost technologies should be seen as the need of the hour, especially when it hardly contributes to public health and patient-related long-term outcomes. Public funds are used to support technologies that remain largely inaccessible to the communities most affected by poor health and financial hardship. It raises fundamental questions concerning fairness, accountability, and the true goals of technology-driven “modernisation” in health care. Healthcare technologies and the Internet-of-Things (IoT) have become integral to digital health and are essential in the present era. One of the main benefits of digital health is its promise to advance health equity through improved access, monitoring, and continuity of care. However, the successful deployment of such digital solutions requires clearly defined outcome targets and context-specific implementation frameworks. Without pre-defined measurable goals and meaningful value at the community level, digital health initiatives risk becoming technology-driven rather than impact-driven. 

The problem is compounded when it is further scrutinised through current scientific evidence. The fact is that many of these technologies deliver only modest additional benefits and are often underutilised [19,20]. PTS can be taken as an illustrative case. While it is promoted as a tool to improve efficiency, hospitals in LMICs frequently struggle with incorporation into existing workflows, maintenance issues, and limited scalability. As a result, utilisation is often inconsistent or well below capacity [19]. Moreover, clinical studies suggest that PTS-based transport does not significantly alter routine blood or coagulation test results, raising uncertainty about its economic efficiency in settings with scarce resources [21]. PTS might reduce turnaround time for laboratories in overcrowded hospitals or emergency areas; evidence that PTS meaningfully improves clinical outcomes remains limited. The evidence of simulation is also poor, especially when compared to patient care-related outcomes. A recent meta-analysis failed to show a difference between simulation-based and non-simulation-based training on patient- and procedure-related outcomes [22]. Simulation offers valuable opportunities for effective visualisation and skills rehearsal, both prior to and alongside in-person teaching. In many LMIC settings, patient volumes per practitioner remain relatively high. Bedside clinical teaching, therefore, continues to represent an efficacious and cost-efficient educational approach. Within this context, the adoption of high-fidelity mannequins and advanced technologies warrants careful appraisal. Decisions should weigh the incremental realism achieved against cost considerations and the potentially limited additional value for routine procedural training, including endoscopy, central venous catheterisation, lumbar puncture, and cannulation. Further, the technology always has room for error. Furthermore, the rapid development of new technologies requires constant retraining of educators and a high cost [23]. Although it mimics the real world, is innovative, provides on-the-go changes, offers multiple options, and allows for repeated misses and errors, even high-fidelity simulation lacks real-world experience and does not guarantee better clinical outcomes. It is unable to fully replicate the complexity and unpredictability of actual clinical scenarios that are faced at the bedside [24]. It is very pertinent, especially when teaching-learning occurs in very dynamic patient situations such as anaesthesia management and mechanical ventilation. This does not imply that simulation-based education lacks value; rather, within LMIC contexts, its adoption may be most impactful when prioritised for life-saving skills training, management of life-threatening complications, and high-risk clinical scenarios, while remaining guided by careful cost appraisal and context-specific educational needs, especially where simpler, inexpensive systems often suffice [25,26]. 

Robotic surgical systems raise even more serious concerns around cost, equity, and sustainability. Surgeons may appreciate the improved ergonomics. Even certain patient subgroups may experience small short-term benefits for select procedures. But these subtle gains come at a very high price. The cost of acquisition, maintenance contracts, proprietary consumables, and staff training adds up quickly [27]. In health systems where OOP payments are common, these additional costs are often transferred directly to patients, increasing the monetary burden of health care [28]. And here is the catch: for many procedures, evidence shows that robotic surgery does not improve survival or provide substantial clinical advantages over conventional laparoscopic or open approaches at the patient's end [29].

In LMICs, the social safety architecture is limited and fragile. Even modest increases in healthcare costs can push families into financial distress. The trade-off is stark: thus, we must sit and ponder such expenditure. Investing heavily in expensive technologies with minimal population-level benefit means fewer resources for essential services that help many. Policy-makers must weigh whether such investments produce value compared to expanding affordable basic care or financial protection for the most vulnerable.

There is another broader, less-discussed issue. The growing influence of the medical technology industry cannot be ignored. Available data suggest that medical device and technology markets in LMICs are booming, driven largely by imports of expensive equipment and aggressive marketing strategies [30,31]. Although direct evidence of corporations' influence on public procurement decisions is limited, broader patterns are evident. In pursuit of hospital or institutional ‎branding, competition for prestige, and the desire to be seen as ‘world-class,’ priorities ‎might shift away from what people actually need or can afford. Strengthening evidence-based management is therefore important. This includes using research to guide decisions, maintaining transparent data practices, improving managerial skills, conducting sound economic evaluations, ensuring responsible cost oversight, and adopting digital systems thoughtfully. Such approaches help keep priority setting focused on patient needs and support fair, accountable use of limited resources [32].

So, when we put all this together and view it through the lenses of distributive justice, opportunity cost, allocative efficiency, and monetary risk, the argument for widespread public financing of high-cost healthcare technologies in most LMIC settings becomes weak. From an allocative efficiency perspective, limited public resources should ideally be directed towards interventions that generate the greatest overall population health gains per unit cost [33]. When healthcare expenditures continue to expose households to financial hardship despite existing protection schemes, prioritising expensive technologies with uncertain population-level impact may inadvertently displace funds for more equitable and cost-effective strategies [33]. In such contexts, careful priority setting that emphasises affordability, scalability, and measurable health outcomes becomes central to maintaining both fiscal sustainability and social accountability [32].

Digital health services and technologies in healthcare are imperative. However, policy should, thus, prioritise real, everyday needs. We should adopt frugal technology models by advancing research and technology towards lean tools and simplified devices, maintaining essential functions for affordability and durability, in place of advanced medical equipment [34]. Digital and telemedicine platforms using low-cost, widely available technology to bridge gaps in care, e.g., India's eSanjeevani platform; contextualised, locally adapted innovations that address specific needs, e.g., Jaipur Foot, 3D printed prosthetics; redesigning procedures to eliminate costly, single-use, or complex equipment in surgical processes, etc., can help us do more with less cost and community-level impact and health equity [34,35]. Low cost for frugal innovations does not mean low impact on patient outcome, but can help us in easing budgetary constraints [36]. The money saved from such a frugal model and context-specific high-cost technology innovations can be invested in health workers, and creating jobs has a lifelong impact on the population and national economy. A mere 5 to 10 crores investment for high-end tech might look minuscule from gross observation, but diverting such an amount can provide a lifetime 15,000-18,000/month job opportunity to 15-25 persons (families) with even inflation adjustment for lifetime (25-30 years of employment) (see the calculation below). Such funds can be used for basic services and for financial protection to prevent CHE. Nevertheless, advanced technologies are not inherently undesirable; they must be adopted only when clear, context-specific value is demonstrated at the patient level, especially for reducing major adverse events, improving long-term outcomes, and providing survival benefits. Otherwise, we risk overvaluing technology at the expense of equity and sustainability, and forgetting who healthcare is for. 

Capital requirement calculation for a 30-year growing expense scenario

Assumptions

- Initial monthly requirement (salary): ₹15,000

- Annual requirement: ₹1,80,000

- Annual investment return (r): 10%

- Annual spending (salary) growth (g): 7.5%

- Time horizon (n): 30 years

- Capital is allowed to be exhausted at the end of 30 years.

Formula Used: Present Value of a Growing Annuity

* *$PV = PMT \times \frac{1 - \left(\frac{1 + g}{1 + r}\right)^{n}}{r - g}$, where PMT = 1,80,000, r = 0.10, g = 0.075, and n = 30. 

Step-by-Step Calculation

Step 1: Computing the ratio: $\frac{1 + g}{1 + r}=\frac{1.075}{1.10} = 0.97727$

Step 2: Raising to the power of 30: $(0.97727)^{30} \approx 0.50$

Step 3: Substituting into the formula: $PV = 180{,}000 \times \frac{1 - 0.50}{0.025} = 180{,}000 \times 20 = 3{,}600{,}000$.

A thirty-year cash flow table for a growing withdrawal (salary) plan is presented in Table 1.

**Table 1:** Thirty-year cash flow table for growing withdrawal (salary) plan Conclusion: To sustain a starting monthly expenditure of ₹15,000 that increases by 7.5% every year, with investments earning 10% annually over a 30-year period, an initial corpus of approximately ₹36 lakh is required. Thus, a 5-10 crore initial capital can provide employment for 13-27 community health workers or multi-tasking workers in tertiary care hospitals. The calculation was done using standard financial modeling, and the table was prepared using a calculator in Microsoft Excel (Microsoft, Redmond, WA, USA).

Year	Opening Balance (₹) (A)	Return Earned (₹) (B)	Withdrawal (Salary) (₹) (C)	Closing Balance (₹) ((A+B)-C)
1	3,600,000	360,000	180,000	3,780,000
2	3,780,000	378,000	193,500	3,964,500
3	3,964,500	396,450	208,012	4,152,938
4	4,152,938	415,294	223,613	4,344,618
5	4,344,618	434,462	240,384	4,538,695
6	4,538,695	453,870	258,413	4,734,151
7	4,734,151	473,415	277,794	4,929,772
8	4,929,772	492,977	298,629	5,124,121
9	5,124,121	512,412	321,026	5,315,507
10	5,315,507	531,551	345,103	5,501,954
11	5,501,954	550,195	370,986	5,681,164
12	5,681,164	568,116	398,810	5,850,471
13	5,850,471	585,047	428,720	6,006,798
14	6,006,798	600,680	460,874	6,146,603
15	6,146,603	614,660	495,440	6,265,824
16	6,265,824	626,582	532,598	6,359,808
17	6,359,808	635,981	572,543	6,423,246
18	6,423,246	642,325	615,483	6,450,087
19	6,450,087	645,009	661,645	6,433,451
20	6,433,451	643,345	711,268	6,365,528
21	6,365,528	636,553	764,613	6,237,468
22	6,237,468	623,747	821,959	6,039,255
23	6,039,255	603,926	883,606	5,759,575
24	5,759,575	575,957	949,877	5,385,656
25	5,385,656	538,566	1,021,117	4,903,104
26	4,903,104	490,310	1,097,701	4,295,713
27	4,295,713	429,571	1,180,029	3,545,256
28	3,545,256	354,526	1,268,531	2,631,250
29	2,631,250	263,125	1,363,671	1,530,705
30	1,530,705	153,070	1,465,946	217,829

## References

[1] (2026). Low-Income Countries Spend Just $17 Per Capita Annually on Health. https://www.worldbank.org/en/news/press-release/2025/11/19/health-financing-challenges-opportunities-changing-aid-landscape-grph#:~:text=New%20World%20Bank%20Group%20Report,%2C%20and%20under%2Dresourced%20clinics..

[2] Homauni A, Markazi-Moghaddam N, Mosadeghkhah A, Noori M, Abbasiyan K, Jame SZ (2023). Budgeting in healthcare systems and organizations: A systematic review. Iran J Public Health.

[3] (2026). World Health Organization: Global Health Expenditure Database: Country Profiles. https://apps.who.int/nha/database/country_profile/index/en.

[4] Wagstaff A, Flores G, Hsu J (2018). Progress on catastrophic health spending in 133 countries: A retrospective observational study. Lancet Glob Health.

[5] Xu K, Evans DB, Kawabata K, Zeramdini R, Klavus J, Murray CJL (2003). Household catastrophic health expenditure: A multicountry analysis. Lancet.

[6] Sriram S, Albadrani M (2022). Impoverishing effects of out-of-pocket healthcare expenditures in India. J Family Med Prim Care.

[7] Kamath S, Maliyekkal J, Elstin Anbu Raj S (2025). Understanding out-of-pocket expenditure in India: A systematic review. Front Public Health.

[8] Srinadh R, Yadav J (2020). Srinadh R, Yadav J. Catastrophic Health Spending and Impoverishment of Households While Seeking Hospital Treatment of Children (0-4 years) in India. NITI Aayog. https://www.niti.gov.in/catastrophic-health-spending-and-impoverishment-households-while-seeking-hospital-treatment.

[9] Ahmad F, Mohanty PC (2024). Incidence and intensity of catastrophic health expenditure and impoverishment among the elderly: An empirical evidence from India. Sci Rep.

[10] National Health Authority (2026). Ayushman Bharat-Pradhan Mantri Jan Arogya Yojana: Operational Guidelines. https://www.nhm.gov.in/New_Updates_2018/NHM_Components/Health_System_Stregthening/Comprehensive_primary_health_care/letter/Operational_Guidelines_For_CPHC.pdf.

[11] (2026). Ministry of Health and Family Welfare. Rashtriya Bal Swasthya Karyakram: Framework for Implementation. New Delhi: Government of India.

[12] Hooley B, Afriyie DO, Fink G, Tediosi F (2022). Health insurance coverage in low-income and middle-income countries: Progress made to date and related changes in private and public health expenditure. BMJ Glob Health.

[13] Chen S, Geldsetzer P, Chen Q (2022). Health insurance coverage in low- and middle-income countries remains far from the goal of universal coverage. Health Aff (Millwood).

[14] Fenny AP, Kusi A, Arhinful DK, Asante FA (2016). Factors contributing to low uptake and renewal of health insurance: A qualitative study in Ghana. Glob Health Res Policy.

[15] Jamison DT, Gelband H, Horton S, Jha P, Laxminarayan R, Mock CN, Nugent R (2017). Disease Control Priorities: Improving Health and Reducing Poverty, 3rd ed. World Bank.

[16] (2026). Healthcare Access in Rural Communities in India - Ballard Brief. https://ballardbrief.byu.edu/issue-briefs/healthcare-access-in-rural-communities-in-india.

[17] Chawla NS (2023). Unveiling the ABCs: Identifying India's healthcare service gaps. Cureus.

[18] Banerjee S (2021). Determinants of rural-urban differential in healthcare utilization among the elderly population in India. BMC Public Health.

[19] Ahidar I, Oubrahim I, Sarsri D, Sefiani N (2024). Reaping benefits and overcoming challenges in pneumatic tube system adoption for hospital logistics: Learnings from a decade of utilization. J Logist Inform Serv Sci.

[20] Goswami G, Sharma SK, Sharma R, Rani R (2021). Simulation and skill training facilities in nursing institutes at Uttarakhand: A cross-sectional study. Iran J Nurs Midwifery Res.

[21] Subbarayan D, Choccalingam C, Lakshmi CK (2018). The effects of sample transport by pneumatic tube system on routine hematology and coagulation tests. Adv Hematol.

[22] Zendejas B, Brydges R, Wang AT, Cook DA (2013). Patient outcomes in simulation-based medical education: A systematic review. J Gen Intern Med.

[23] Jeet G, Prinja S, Aggarwal AK (2017). Cost analysis of a simulation-based training for health workforce in India. Indian J Public Health.

[24] Elendu C, Amaechi DC, Okatta AU, Amaechi EC, Elendu TC, Ezeh CP, Elendu ID (2024). The impact of simulation-based training in medical education: A review. Medicine (Baltimore).

[25] Zendejas B, Wang AT, Brydges R, Hamstra SJ, Cook DA (2013). Cost: the missing outcome in simulation-based medical education research: A systematic review. Surgery.

[26] Barbash GI, Glied SA (2010). New technology and health care costs--The case of robot-assisted surgery. N Engl J Med.

[27] (2026). The Literature on Health Care Simulation Education: What Does It Show?. https://psnet.ahrq.gov/perspective/literature-health-care-simulation-education-what-does-it-show.

[28] Lai TJ, Roxburgh C, Boyd KA, Bouttell J (2024). Clinical effectiveness of robotic versus laparoscopic and open surgery: An overview of systematic reviews. BMJ Open.

[29] Duhoky R, Rutgers ML, Burghgraef TA (2024). Long-term outcomes of robotic versus laparoscopic total mesorectal excisions: A propensity-score matched cohort study of 5-year survival outcomes. Ann Surg Open.

[30] (2026). World Health Organization. Medical Devices: Managing the Mismatch—An Outcome of the Priority Medical Devices Project. https://iris.who.int/server/api/core/bitstreams/7d762e5a-c35a-4606-ae55-7e43c3f49156/content.

[31] (2026). International Trade Administration. India Surgical Robotics Market Intelligence Report. Department of Commerce.

[32] Sallam DM, Stanley DA, Snygg J, Jelley D, Sajwani A (2025). Empowering healthcare leadership through facilitators of evidence-based management: A narrative review and proposed conceptual framework. Front Manag Sci.

[33] Ochalek J, Lomas J, Claxton K (2018). Estimating health opportunity costs in low-income and middle-income countries: A novel approach and evidence from cross-country data. BMJ Glob Health.

[34] Sarkar S, Mateus S (2022). Doing more with less - How frugal innovations can contribute to improving healthcare systems. Soc Sci Med.

[35] Tran VT, Ravaud P (2016). Frugal innovation in medicine for low resource settings. BMC Med.

[36] (2023). Frugal innovation: Why low cost doesn't have to mean low impact. Nature.

